# Fungal Genetics and Functional Diversity of Microbial Communities in the Soil under Long-Term Monoculture of Maize Using Different Cultivation Techniques

**DOI:** 10.3389/fmicb.2018.00076

**Published:** 2018-01-30

**Authors:** Anna Gałązka, Jarosław Grządziel

**Affiliations:** Department of Agricultural Microbiology, Institute of Soil Science and Plant Cultivation - State Research Institute, Puławy, Poland

**Keywords:** fungal community, genetic diversity, metabolic profiles, biolog ecoplates, ITS-1 NGS

## Abstract

Fungal diversity in the soil may be limited under natural conditions by inappropriate environmental factors such as: nutrient resources, biotic and abiotic factors, tillage system and microbial interactions that prevent the occurrence or survival of the species in the environment. The aim of this paper was to determine fungal genetic diversity and community level physiological profiling of microbial communities in the soil under long-term maize monoculture. The experimental scheme involved four cultivation techniques: direct sowing (DS), reduced tillage (RT), full tillage (FT), and crop rotation (CR). Soil samples were taken in two stages: before sowing of maize (DS_BS_-direct sowing, RT_BS_-reduced tillage, FT_BS_-full tillage, CR_BS_-crop rotation) and the flowering stage of maize growth (DS_F_-direct sowing, RT_F_-reduced tillage, FT_F_-full tillage, CR_F_-crop rotation). The following plants were used in the crop rotation: spring barley, winter wheat and maize. The study included fungal genetic diversity assessment by ITS-1 next generation sequencing (NGS) analyses as well as the characterization of the catabolic potential of microbial communities (Biolog EcoPlates) in the soil under long-term monoculture of maize using different cultivation techniques. The results obtained from the ITS-1 NGS technique enabled to classify and correlate the fungi species or genus to the soil metabolome. The research methods used in this paper have contributed to a better understanding of genetic diversity and composition of the population of fungi in the soil under the influence of the changes that have occurred in the soil under long-term maize cultivation. In all cultivation techniques, the season had a great influence on the fungal genetic structure in the soil. Significant differences were found on the family level (*P* = 0.032, *F* = 3.895), genus level (*P* = 0.026, *F* = 3.313) and on the species level (*P* = 0.033, *F* = 2.718). This study has shown that: (1) fungal diversity was changed under the influence different cultivation techniques; (2) techniques of maize cultivation and season were an important factors that can influence the biochemical activity of soil. Maize cultivated in direct sowing did not cause negative changes in the fungal structure, even making it more stable during seasonal changes; (3) full tillage and crop rotation may change fungal community and soil function.

## Introduction

Research on the biological diversity of soil microorganisms is concentrated on three aspects of diversity: species, genetic and functional (Bundy et al., 2009; Bowles et al., 2014). In biodiversity analysis, it is important to evaluate the microbiome as a whole, not only its individual components (Daghino et al., 2012). Research on microbial activity in different environments is essential to increase knowledge about the ecology of their biocenosis and should be analyzed in connection with the existing environmental conditions, considering both biotic and abiotic factors (Bowles et al., 2014). Undoubtedly, an important aspect of such research is the selection and development of appropriate indicators and methods for assessing soil biodiversity and the activity of soil microorganisms, so that they can give the most reliable and reproducible results (Brussaard et al., 2007; Ghimire et al., 2014).

Investigation of soil biological activity as one of the indicators in the evaluation of tillage systems for the needs of sustainable agriculture was undertaken. It has been assumed that through the elaboration of the correct cropping technique, it will be possible to significantly increase the degree of fungal and bacterial diversity and reducing loss soil biodiversity (Danielsen et al., 2012).

Additionally, the introduction of such a cropping system should result in the reduction of energy consumption and labor-intensive tillage (Brussaard et al., 2007). The cultivation of plants in monoculture may be such a system. Tillage practices and cultivation techniques as well as residue management have an important effect on biological activities and the functional diversity of microorganisms (Lupwayi et al., 1998). Monoculture of plants can induce important changes in the soil environment and biological activity related to the reduction of fungal diversity (Liang et al., 2011). Many authors have suggested that long-term cropping of plants in monoculture induces degradation processes in the soil that can lead to a reduction in the a number of fungal species and a decline in organic matter (Brussaard et al., 2007; Liang et al., 2011; Han et al., 2017). Maize cultivation in monoculture is practiced in many countries. The cultivation of maize in direct sowing (zero-tillage) in Polish conditions is a good alternative but requires further systematic research on interactions between system factors: biotic and abiotic factors, soil environment, plants and fungi (Gałązka et al., 2017a,b). Maize (*Zea mays* L.) is one of the most important crops and is widely used in agriculture and industry, but its cultivation is very energy-intensive, hence agricultural practice is looking for a simpler solution (Liang et al., 2011; Gałązka et al., 2017c). The cultivation of some plants such as maize in a zero-tillage system is the most attractive and gives the biggest economic profits. Maize is grown increasingly in direct sowing and in this system leaves many crop residues are left on the surface of the field (Zhang et al., 2012).

The effects of long-term monoculture on soil quality, especially fungal genetic diversity, are not widely recognized for cultivation. Research on the effects of long-term cultivation of maize in monoculture on changes in soil quality, vegetation and yield of maize has been conducted at IUNG (Institute of Soil Science and Plant Cultivation, State Research Institute, Pulawy, Poland) for many years (Gałązka et al., 2017a,c). Long-term monoculture has a strong influence on soil parameters, especially on soil microorganisms and enzymes.

However, it is not known how the use of different techniques for cultivating maize growth may influence the composition of the fungal community. Biochemical and microbial properties of soil respond very rapidly to even small changes in environmental factors, such as temperature, moisture, tillage or cultivation techniques, and may have a strong impact on the core microbiome of fungi (Liang et al., 2011; Zhang et al., 2012). The term “core microbiome of fungi” can be understood as the group of fungi comprised of the members common to two or more fungal assemblages associated with a habitat (Shade and Handelsman, 2011). Evaluating the core fungal species is essential to unraveling the ecology of fungi consortia. Hence, bacterial and fungal communities are very often used as early indicators of soil alteration induced by agricultural management (Nannipieri, 2011).

Fungi constitute the biodiversity group agricultural soil and perform numerous important ecosystem functions such as influencing plant health (Wang et al., 2017). Fungal biodiversity in soil has been increasingly recognized as being beneficial for soil health (Fisher et al., 2012; Duniere et al., 2017). In current research on fungal biodiversity, a very important issue is not only the identification and distribution of this group, but also the definition of their important roles in ecosystems (Fisher et al., 2012). Comparison of the functional diversity of soil and the genetic diversity of fungi may enable a better understanding of their fundamental and ecological role and impact on plant health. Several environmental factors, including the physicochemical properties of soil, biological activities, soil moisture, biomass carbon and nitrogen, organic matter content, climate, season and also tillage systems, may significantly impact the diversity of the fungal genetic community in the soil environment (Liang et al., 2011).

The functional and structural diversity of soil fungi have been evaluated using several parameters, such as microbial biomass, respiration and enzymatic activities, as well as molecular methods, including next-generation sequencing (Lim et al., 2010; Xu, 2010; Zhang et al., 2012; Welc et al., 2014; Wang et al., 2017). Next-generation sequencing (NGS) of the hyper -alternating regions (16S rDNA for bacteria and ITS for fungi) enables definition of the genetic diversity of microorganisms without cell culture cultivation (Kozich et al., 2013; Orgiazzi et al., 2013). It relies on the isolation of total DNA from soil samples and preliminary amplification of hyper-alternating regions with the use of specific starters. In the next stage, correct adaptors and indexes are attached to amplicons (Ranjard et al., 2003; O'Brien et al., 2005; Schoch et al., 2012; Orgiazzi et al., 2013). The concentrations of samples are normalized and then all samples are combined into one cumulative sample that is designated for sequencing. The use of adequate indexes allows individual amplicons to be assigned to appropriate samples (Bartram et al., 2011; Kozich et al., 2013; Zoll et al., 2016).

In current paper, we propose that more insight should be gained into the determination of fungal structural diversity and community level and physiological profiling of microorganisms in the soil under long-term maize monoculture. The other objective of this work was to identify which groups of soil fungi are most sensitive to these techniques. Application of both genetic and functional methods will allow investigation of the composition of the fungal community and functionality of soil microorganisms directly in the soil. It will also allow explanation of the possible relationships between fungi and microbial community with using cultivation techniques.

## Materials and methods

### Field experiment

The study was based on in the a long—term stationary field experiment. The soil in this experiment was classified as gray brown podsolic soil formed from light loam (USDA: SiL silt loam). This field experiment was established in 2004 at the Institute of Soil Science and Plant Cultivation's Agricultural Experimental Station (AES) in Grabow, Mazowieckie Voivodship (51°23' N; 21°38' E), Poland. The experimental scheme involved four cultivation techniques: direct sowing (DS), reduced tillage (RT), full tillage (FT), and crop rotation (CR). Soil samples were taken twice a year: before sowing of maize (the index bottom BS): DS_BS_-direct sowing, RT_BS_-reduced tillage, FT_BS_-full tillage, CR_BS_-crop rotation and in the flowering stage of maize growth (the index bottom F): DS_F_-direct sowing, RT_F_-reduced tillage, FT_F_-full tillage, CR_F_-crop rotation. The following plants were used in the crop rotation: spring barley, winter wheat and maize. The field experiment was carried out with the long strips with the mirror image of combinations. More information about the character and plan of this experiment can be found in Gałązka et al. (2017c).

In the full tillage technique, straw residues were left after the cob harvest, then shredded, and turned under (Gałązka et al., 2017c). By contrast, in the direct sowing the straw was shredded but left on the soil surface. Under the crop rotation management, all the crop species involved were grown each year and full doses of fertilization and herbicides were applied to maize. Maize cv. Delitop was seeded using a precision maize planter. Nitrogen was applied to the maize at a rate of 140 kg N ha^−1^ (70 + 70), phosphorus and potassium rates (kg·ha^−1^) were P_2_O_5_ – 80 and K_2_O – 125. Annual fertilizer rates applied to barley were: N – 60, P_2_O_5_ – 35 and K_2_O – 50 kg ha^−1^, and to wheat: N – 120, P_2_O_5_ – 40 and K_2_O – 70 kg·ha^−1^. The results of physicochemical properties and soil quality have already been published (Gałązka et al., 2017a,c).

### Soil samples

Soil samples were collected in 2016 according to (Polish Standard PN-ISO 10381-6, 1998) in two sampling times: before sowing and in the flowering phase of maize growth. The soil samples in three replicates were taken from a 0–30 cm layer (as bulk soil samples from a given field), sieved through a 2 mm sieve and stored in a refrigerator (4°C) until analysis.

### Community level physiological profiling (CLPP) analysis using biolog ecoplates

The metabolic potential of soil microbial communities was evaluated using Biolog EcoPlate (Biolog Inc., Hayward, CA, USA). The metabolic capacities of all soil microorganisms (bacteria and fungi together) were determined using the EcoPlates system with 31 different carbon sources. Soil suspension for the inoculation of wells in microplates was prepared as follows: 1 g of soil was weighed, transferred to conical flasks holding 99 cm^3^ sterile 0.9% NaCl each, and vortexed for 30 min at 150 rpm and at 25°C, after which the samples were cooled for 30 min to 4°C (Pohland and Owen, 2009). After that, 120 mm^3^ was transferred to each of the wells in an EcoPlate and incubated in the dark at 28°C for 168 h. The experiment included three replications. The results were read on the MicroStation ID system by the Biolog®.

The extent to which carbon sources were used was determined through the reduction of colorless tetrazolium chloride to red formasane (λ = 490 nm) (Insam and Goberna, 2004). The intensity of color development was recorded at λ = 490 nm for a period of 168 h at 24-h intervals. The most intensive metabolism of carbon substrates was observed after 120–168 h of incubation, but the results obtained at 168 h are presented in the paper. The activities of soil microorganisms are based on all carbon sources and on grouped sources defined as amines and amides, amino acids, carbohydrate, carboxylic acid and polymers (Pohland and Owen, 2009). The results were expressed as Average Well-Color Development (AWCD) and Shannon-Weaver (*H*') indices.

### DNA extraction, ITS-1 next- generation sequencing (NGS) and bioinformatics analyses

Total DNA was extracted from 0.5 g of soil using a FastDNA® SPIN Kit for Soil (MP Biomedicals, OH, USA), according to the manufacturer's instructions. A NanoDrop 2000 spectrophotometer (Thermo Scientific, USA) was used to determine the concentration and quality of the DNA. The fungal internal transcribed spacer-1 (ITS-1) region was amplified from each sample using primers ITS1FI2 (5′-GAACCWGCGGARGGATCA-3′) and 5.8S (5′-CGCTGCGTTCTTCATCG-3′), which provide a comprehensive coverage with the highest taxonomical accuracy for fungal sequences (Mello et al., 2011; Orgiazzi et al., 2013; Schmidt et al., 2013). The PCR was performed using Q5 Hot Start High-Fidelity 2x Master Mix, with reaction conditions according to the manufacturer's recommendations. The reaction was carried out according to the Illumina ITS-1 amplification protocol and sequencing was performed on an Illumina MiSeq (Genomed S.A., Warsaw, Poland). The reverse primer contained a 8-bp error-correcting barcode, unique to each sample. The libraries were prepared in analogously to the attached Illumina protocol. Sequencing was performed on a MiSeq by Illumina Inc. using paired—end (PE) technology, with 2 × 250 cycles with v2 chemistry, according to the manufacturer's recommendations. Automatic preliminary data analyses were performed using an MiSeq Reporter (MSR) v2.6. These analyses consisted of following stages: adaptor sequences trimming— program cutadapt, quality control and trimming of low quality bases (quality < 20, min length 30)—program cutadapt, paired reads joining—fastq-join algorithm, OTU clustering with 97% sequence similarity—uclust algorithm, chimeras detection and removal—usearch61 algorithm and taxonomy assignment based on UNITE v7 database—blast algorithm.

Bioinformatics analyses, including classification of reads to species level, were performed using QIIME (Quantitative Insights Into Microbial Ecology) based on the reference databases UNITE v7 (Caporaso et al., 2010). Cluster generation was based on the chosen database of reference sequences, removal of chimer sequences and attribution of taxonomy. The results are presented in OTU (Operational Taxonomic Units) containing the classification and number of reads in every OTU in BIOM (Biological Observation Matrix) format.

### Statistical analysis

The main statistical analyses were performed using STATISTICA.PL (10) (Stat. Soft. Inc. USA). The data was subject to a three-way (fungal community, cultivation techniques, biodiversity indices from EcoPlate) analysis of variance (ANOVA) for the comparison of means. Significant differences were calculated according to Tukey's *post-hoc* HSD test at significance level *P* < 0.05. The Average Well-Color Development (AWCD) was evaluated according to (Garland and Mills, 1999) in with formula AWCD = Σ (C-R)/95; where C = the absorbancy in each well and R = the absorbancy in the control well. The Shannon—Weaver (*H'*) index was evaluated in accordance with the formula *H'* = −Σpi(lnpi), where pi = the ratio of the absorbance of each well to the absorbance of all wells (Gomez et al., 2004). The results were also submitted to the PC (principal component) analysis in order to determine common relations between the fungal community and the soils collected using different cultivation techniques. For the principal coordinate analysis (PCoA) and Welch's test, the data was expanded using statistical analyses of fungal community profiles (STAMP 2.1.3) software (Parks et al., 2014). This analysis was employed to study statistically significant differential abundance of different-level taxa among soil fungal community. The results were calculated as each taxon-relative abundance, assuming all assigned reads per sample to be 100%. Welch's test (two-group analysis) was performed applying a *t*-test with 95% confidence intervals. Only taxonomic representatives that differed significantly (*P* < 0.05) among different soils were taken into account. Principal coordinate analysis (PCoA) was performed at the species level, using Euclidean distance measurements. Permutational multivariate analysis of variance (PERMANOVA) was used to compare the microbial community structure between soils taken from different sites and with different contamination times. This was performed with 999 permutations using the Adonis function of the PAST package (v 3.16) (Hammer et al., 2001).

## Results

### Community level physiological profiling (CLPP) of soil

**Figure 1:**
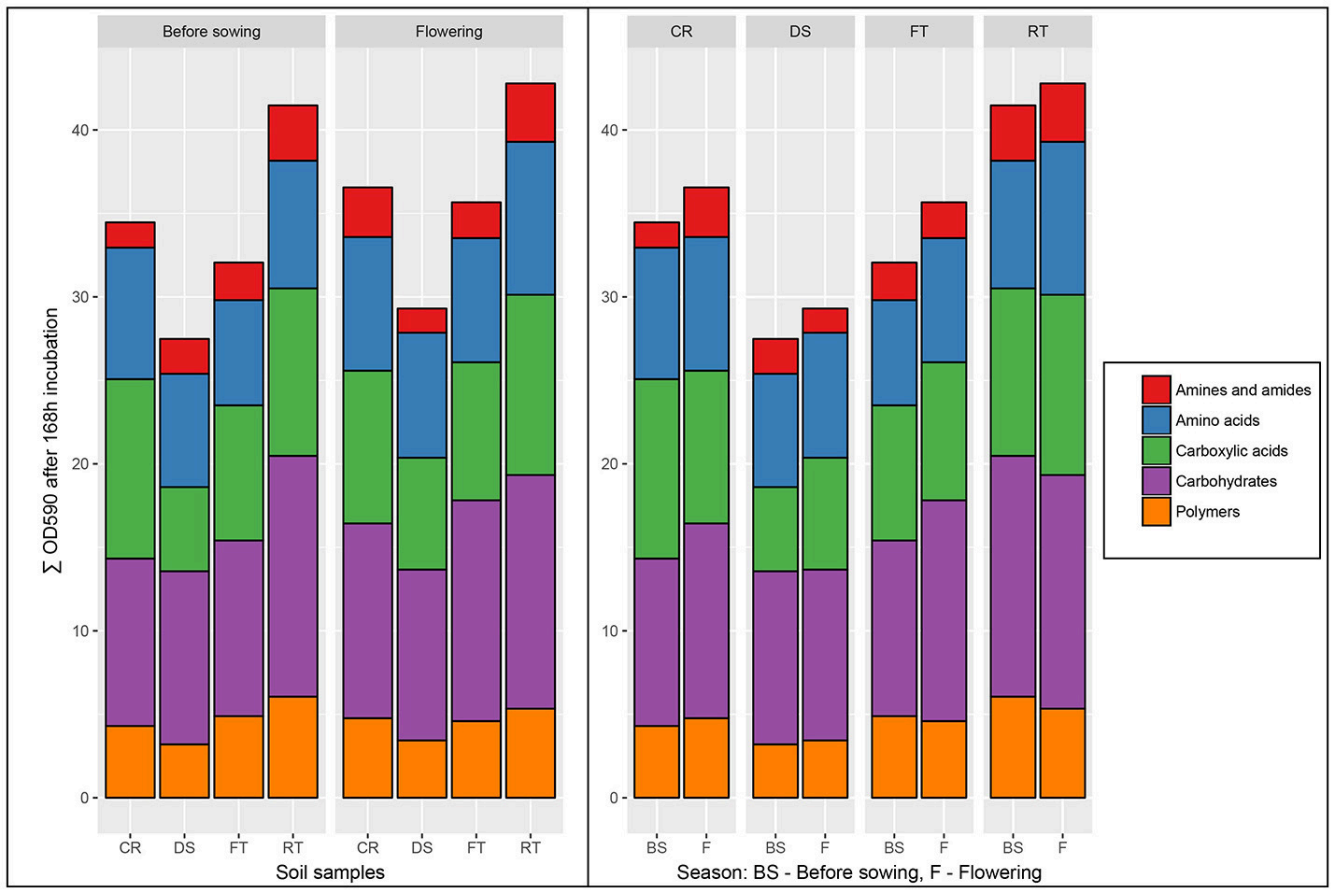
The microbial community catabolic diversity in the soil collected from monoculture of maize according to Biolog EcoPlates incubated for 168 h. Treatment means separated by different letters are significantly different (*P* < 0.05). Soil collected before maize sowing: DS_BS_, direct sowing; RT_BS_, reduced tillage; FT_BS_, full tillage; CR_BS_, crop rotation. Soil collected in flowering stage of maize growth: DS_F_, direct sowing; RT_F_, reduced tillage; FT_F_, full tillage; CR_F_, crop rotation.

The effects of seasons and cultivation techniques on microbial community catabolic diversity as evaluated by main substrate utilization in the Biolog EcoPlate, were measured (Figure 1). The soil samples collected before the sowing of maize—DS_BS_, RT_BS_, FT_BS_, and CR_BS_ were characterized by statistically lower indexes of biological activity of substrate utilization than the soil collected in the flowering stage of maize growth (DS_F_, RT_F_, FT_F_, CR_F_). This effect was present for all the evaluated substrate groups: amines and amides, carboxylic and acetic acids, carbohydrates, polymers, aminoacids and for the percent of total carbon source utilization in the soil. Also the effects of seasons and cultivation techniques on microbial community catabolic diversity was presented as the radar plot (Figure S1).

**Figure 2:**
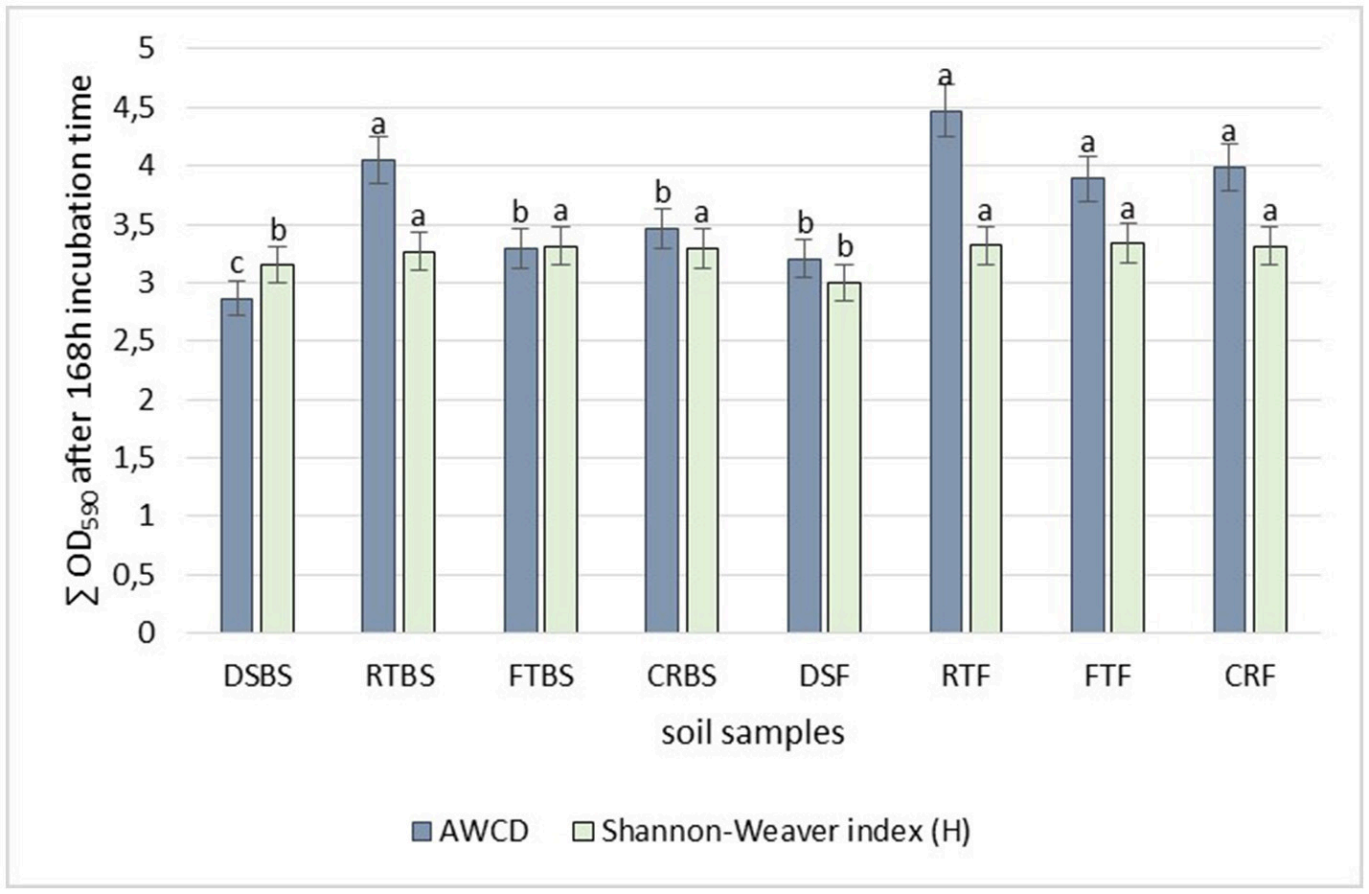
Effect of different cultivation techniques on microbial community catabolic diversity as evaluated by: the Shannon's diversity index (*H*) and average well-color development (AWCD_590_) in the Biolog EcoPlate incubated for 168 h (*P* < 0.05). Soil collected before maize sowing: DS_BS_, direct sowing; RT_BS_, reduced tillage; FT_BS_, full tillage; CR_BS_, crop rotation; Soil collected in flowering stage of maize growth: DS_F_, direct sowing; RT_F_, reduced tillage; FT_F_, full tillage; CR_F_, crop rotation. Treatment means separated by different letters are significantly different (*P* < 0.05).

The soil samples collected before maize sowing from full tillage as the cultivation technique (FT_BS_) were characterized by higher community level physiological profiling than the soils obtained in the maize flowering stage (FT_F_). The highest diversity based on the Shannon-Weaver index was found in the soil from full tillage in both the before sowing and at flowering stages of maize growth (FT_BS_, *H'* = 3.31 and FT_F_, *H'* = 3.34) (Figure 2). Also, a higher diversity was found in the soil cultivated using reduced tillage from the flowering stage (RT_F_, *H'* = 3.29) and crop rotation (CR_F_, *H'* = 3.31) (Figure 2) than in other tillage systems. The lowest diversity was recorded for the soil collected before sowing from direct sowing fields (DS_BS_, *H'* = 3.14).

The soils obtained in the flowering stage of maize cultivated using different sowing techniques were also characterized by a higher Average Well-Color Development (AWCD) index (Figure 2). The highest activity was found in the soil samples from reduced tillage (RT_F_, AWCD = 4.47), followed by full tillage (FT_F_, AWCD = 3.89) and crop rotation (CR_F_, AWCD = 3.98). The lowest AWCD index was found in the soil from direct sowing (zero tillage) collected before the sowing of maize (DS_BS_, AWCD = 3.14).

### ITS-1 next-generation sequencing

The taxonomic composition was determined based on the relative abundances of dominant class, orders, genera, and species. Fungi abundances were exported as a representative hit classification to avoid inflated hit counts, in accordance with the MG-RAST manual (ftp://ftp.metagenomics.anl.gov/data/manual/mg-rast-manual.pdf), section 4.5. Sequences and corresponding detailed analysis parameters are deposited in the MG-RAST server under sample identification numbers (Table 1). In addition, the raw data was also deposited in The European Nucleotide Archive (ENA) (ID project: PRJEB24318; http://www.ebi.ac.uk/ena/data/view/PRJEB24318 (Table 1). The classification rate summary (at least 97% sequence similarity) for all the analyzed soil samples is presented in Supplementary Materials (Table S1). The highest classification rate summary (at least 97% sequence similarity) was found for the soil sample RT_BS_ (the soil before sowing, reduced tillage) and equalled 237924 read classified, which constituted 72.38%. The lowest classification rate summary (at least 97% sequence similarity) was found for the soil samples: CR_BS_ (soil before sowing, crop rotation) and DS_BS_ (crop rotation) and equalled 60746 read classified for the CR_BS_ sample or 44.01%, and 162114 reads classified for the r DS_BS_ sample. The table of main species along with the full name according to Index Fungorum was included in Supplementary Materials (Table S2).

**Table 1:** Summary of deposited sequencing data.

**Sample ID**	**Post-QC Sequences count**	**Post-QC Mean sequence length**	**Post-QC mean GC percent(%)**	**MG-RAST ID**	**ENA ID**
DS_BS_	61.095	255 ± 72 bp	51 ± 10	mgm4755961.3	ERS2075820
RT_BS_	62.993	252 ± 70 bp	49 ± 9	mgm4755954.3	ERS2075821
FT_BS_	54.558	258 ± 72 bp	50 ± 10	mgm4755955.3	ERS2075822
CR_BS_	65.230	227 ± 72 bp	50 ± 10	mgm4755958.3	ERS2075823
DS_F_	63.466	251 ± 68 bp	50 ± 10	mgm4755959.3	ERS2075824
FT_F_	36.370	222 ± 74 bp	50 ± 10	mgm4755957.3	ERS2075825
FT_F_	40.192	244 ± 65 bp	52 ± 9	mgm4755956.3	ERS2075826
CR_F_	62.567	255 ± 71 bp	50 ± 10	mgm4755960.3	ERS2075827

*Public data is available in the MG-RAST database (http://metagenomics.anl.gov/linkin.cgi?project=mgp81449); number of project: mgp81449. In addition, the raw data was also deposited in The European Nucleotide Archive (ENA) (ID project: PRJEB24318; http://www.ebi.ac.uk/ena/data/view/PRJEB24318. Soil collected before sowing: DS_BS_, direct sowing; RT_BS_, reduced tillage; FT_BS_, full tillage; CR_BS_, crop rotation. Soil collected in flowering stage of maize growth: DS_F_, direct sowing; RT_F_, reduced tillage; FT_F_, full tillage; CR_F_, crop rotation*.

Significant differences in the fungal community structure were found between soil taken from different sampling time (before the sowing and flowering stages of maize growth). These significant differences were found on the family level (PERMANOVA, *P* = 0.032, *F* = 3.895), genus level (PERMANOVA, *P* = 0.026, *F* = 3.313, and on the species level (PERMANOVA, *P* = 0.033, *F* = 2.718).

**Figure 3:**
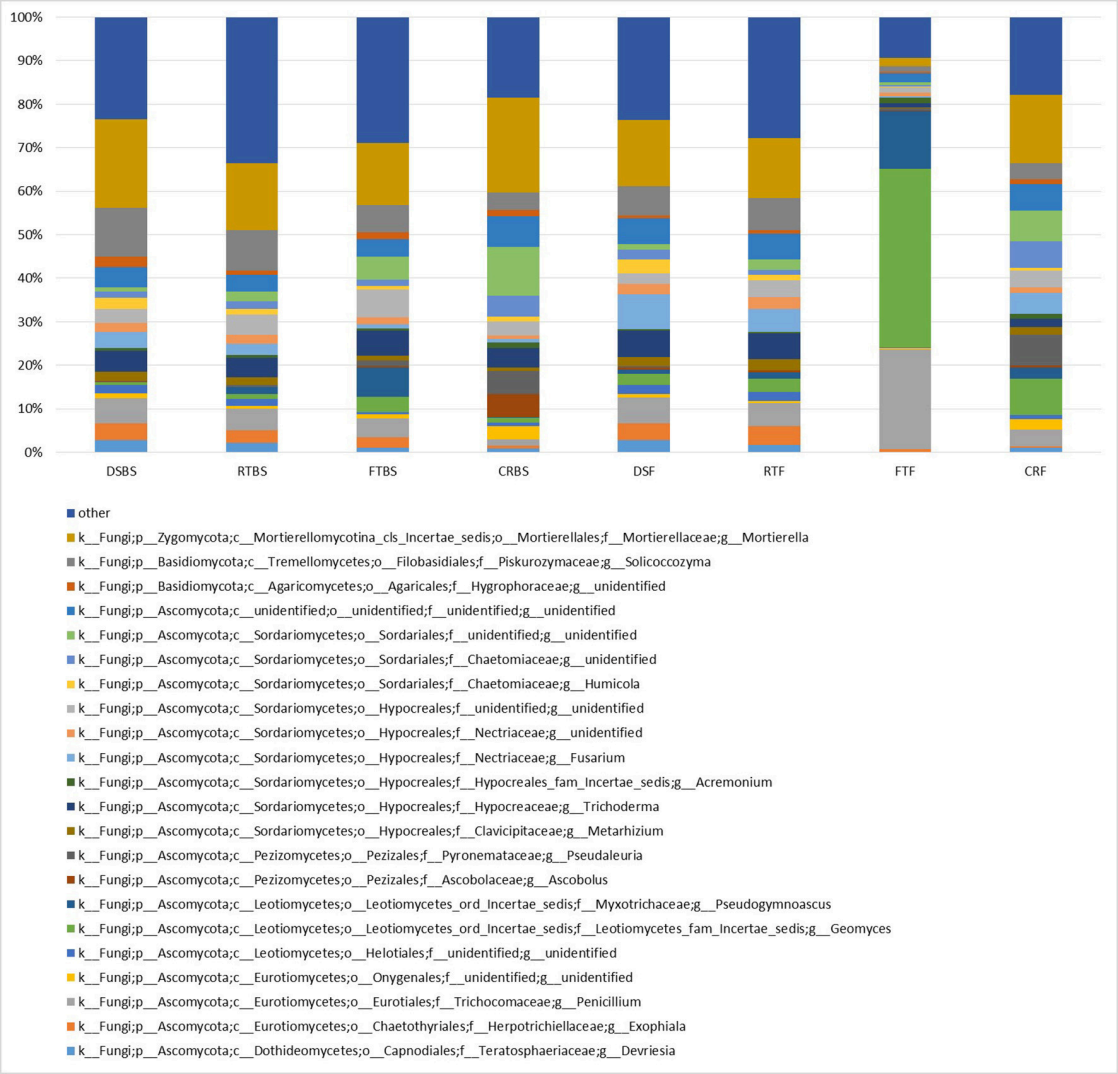
The ITS-1 next generation sequencing for fungal genus. The classifications with less than 1% abundance are gathered into the category “other”.

Furthermore, the allocation of the assembled contig sequences to fungal genome sequences based on ITS-1 next-generation sequencing for fungal genus sequences is presented in Figure 3. The main group among the fungi accounted for three phyla: *Zygomycota, Basidiomycota*, and *Ascomycota*. *Ascomycota* was the dominant phylum and was identified in all the analyzed soil samples (Figure 3). Six dominant fungal classes were also identified in the soils: *Dothideomycetes, Eurotiomycetes, Leotiomycetes, Pezizomycetes, Tremellomycetes*, and *Mortierellomycotina*. The significant differences in the genetic structure of the fungal community were observed in the soil collected from full tillage in summer in the flowering stage of maize (FT_F_) (Figure 3). The dominant genera in this soil obtained in basic relative abundance were: *Penicillum* (28.3%), *Geomyces* (18.4%), *Mortierella* (12.3%), and *Pseudogymnoascus* (11.8%). Significant differences based on the allocation of the assembled contig sequences to fungal community were observed in the fungal genetic structure of soils collected before the sowing and flowering stages of maize growth. Also, the soil collected from full tillage fields was characterized by a different genus structure compared to direct sowing and other cultivation techniques. Soil collected from direct sowing of maize was characterized by the most stable fungal genetic structure, independing on the season compared with other cultivation techniques (Figure 3).

Correlation of the ITS fungal genera with the first (PC1) and second (PC2) components of principal components analyses is presented in Table 2 (statistically significant at *P* ≤ 0.05). The following genera were revealed in the analyzed soils: *Alternaria, Bionectria, Boeremia, Chaetomium, Cladosporium, Cochliobolus, Cylindrocarpon, Davidiella, Emericellopsis, Epichloe, Epicoccum, Fusarium, Gibberella, Glomerella, Hypocrea, Lecanicillium, Lewia, Paecilomyces, Peyronellaea, Phoma, Trichoderma, Verticillium, Conocybe, Cryptococcus, Guehomyces, Sporobolomyces, Olpidium, Mortierella, Mucor*, and *Zygorhynchus*.

**Table 2:** Correlation of ITS fungal genera with the first (PC1) and second (PC2) component (statistically significant (*P* ≤ 0.05).

**Genus**	**PC1 (33.23%)**	**PC2 (18.96%)**
*Alternaria*	−0.556	
*Bionectria*		0.605
*Boeremia*	−0.754	−0.554
*Chaetomium*		0.747
*Cladosporium*		0.763
*Cochliobolus*	−0.680	
*Cylindrocarpon*	−0.695	−0.616
*Davidiella*		0.641
*Emericellopsis*		−0.792
*Epichloe*	−0.695	−0.616
*Epicoccum*		0.824
*Fusarium*	−0.921	
*Gibberella*	−0.826	0.517
*Glomerella*	−0.900	
*Hypocrea*	−0.693	
*Lecanicillium*	−0.868	
*Lewia*	−0.733	
*Paecilomyces*	−0.785	
*Peyronellaea*	−0.695	−0.616
*Phoma*		−0.597
*Trichoderma*	−0.689	−0.522
*Verticillium*		0.733
*Conocybe*	−0.520	0.670
*Cryptococcus*	−0.538	
*Guehomyces*	−0.520	0.670
*Sporobolomyces*		0.725
*Olpidium*	−0.695	−0.616
*Mortierella*	−0.731	
*Mucor*	−0.879	
*Zygorhynchus*		0.822

**Figure 4:**
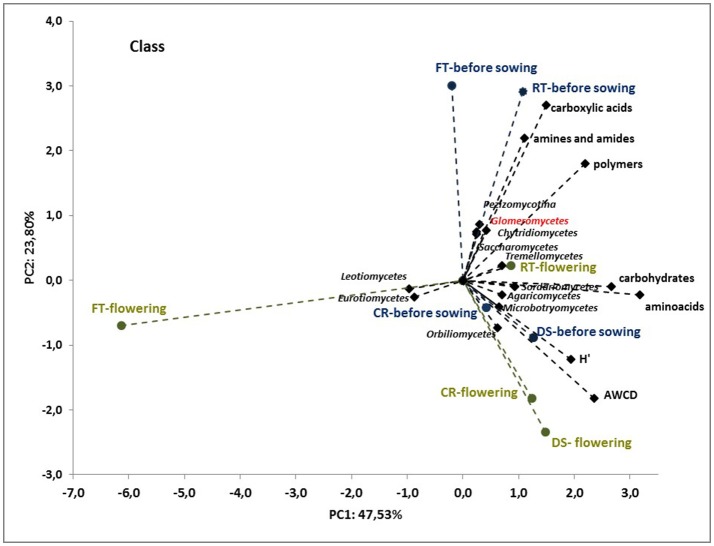
Principal component analysis (PC) of ITS fungal class community and the Shannon's diversity index (*H*) and average well-color development (AWCD_590_) in the Biolog EcoPlate incubated for 168 h (*P* < 0.05).

**Figure 5:**
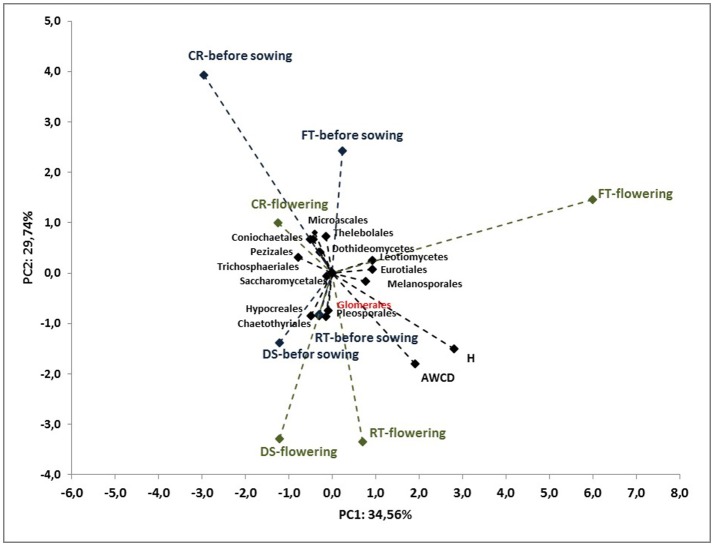
Principal component analysis (PC) of ITS fungal orders community and the Shannon's diversity index (*H*) and average well-color development (AWCD_590_) in the Biolog EcoPlate incubated for 168 h (*P* < 0.05).

**Figure 6:**
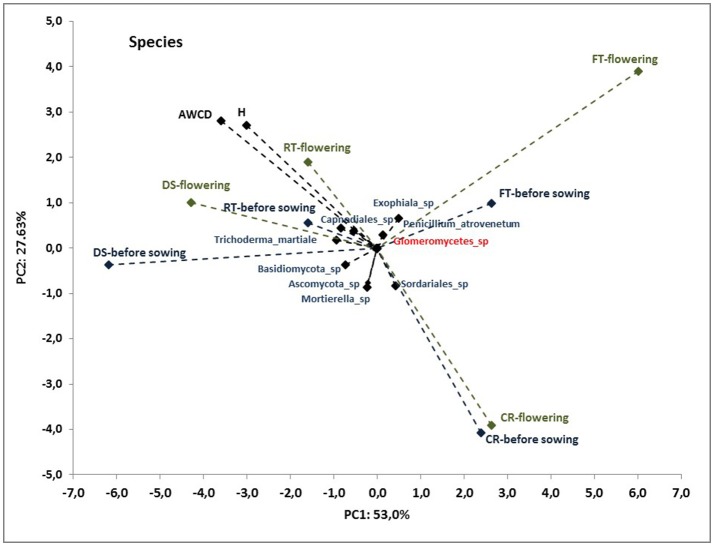
Principal component analysis (PC) of ITS fungal species community and the Shannon's diversity index (*H*) and average well-color development (AWCD_590_) in the Biolog EcoPlate incubated for 168 h (*P* < 0.05).

**Figure 7:**
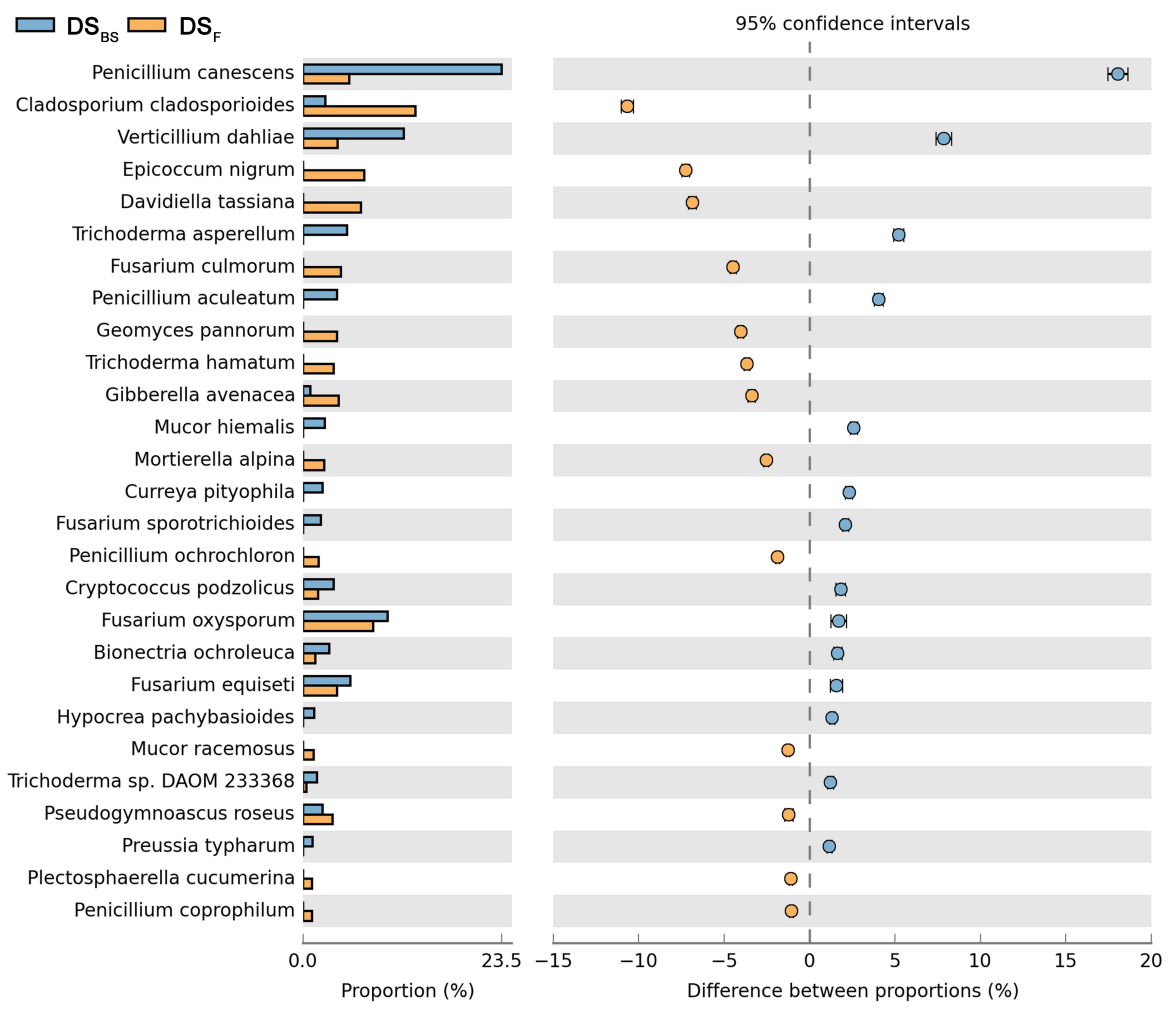
Comparison of fungal species composition depending on the season: before sowing, flowering stage and direct sowing. The table of main species along with the full name according to **Index Fungorum** was included in Supplementary Materials (Table S1).

To better understand the interdependence and correlation the fungal community structure, functional diversity of microbial community, different cultivation techniques and seasons, a biplot of principal component analysis (PC) was obtained. This analysis was performed for selected phylogenetic levels: class (Figure 4), order (Figure 5) and species (Figure 7). Based on biplot PC for fungal classes, the soils were grouped as follows: soils collected from full tillage from the flowering stage of maize growth (FT_F_) with two dominant classes: *Leucoimycetes* and *Eurotiomycetes*, and the second group: soil collected before the sowing from full tillage (FT_BS_) and reduced tillage (RT_BS_). A third group was allocated to soils from other cultivation techniques with dominant classes: *Sacharomycetes, Sordariomycetes, Agariomycetes, Microbotrymycetes, Tremellomycetes, Pezizomycotina*, and *Glomeromycetes* (Figure 4). On the other hand, based on biplot PC for fungal orders, the soils were divided into three groups (Figure 5). The first group comprised soils collected from direct sowing (DS_BS_ and DS_F_) and reduced tillage (RT_BS_ and RT_F_) with the following dominant orders: *Pleosporales, Glomerales, Hypocreales, Chaetothyriales*. The second group consists of soils collected from full tillage before sowing (FT_BS_) and soils collected from crop rotation (CR_BS_ and CR_F_) with the following dominant orders: *Thelebolales, Pezizales, Saccharomycetales, Coniochaetales*. The third group was soil collected from full tillage from the flowering stage of maize growth with the dominant orders *Eurotiales, Leotiomycetes*, and *Melanosporales* (Figure 5). Based on biplot PC for fungal species the soils were divided into three groups but this clustering was strictly conditioned in terms of cultivation techniques (Figure 6). The first group were soils collected from direct sowing (DS_BS_ and DS_F_) and reduced tillage (RT_BS_ and RT_F_) with the following dominant species: *Ascomycota* sp., *Basidiomycota* sp., *Trichoderma martiale, Glomeromycetes* sp., *Capnodiales* sp., and *Mortierella* sp. The second group comprised soils collected from full tillage (FT_BS_ and FT_F_) with dominant species *Penicillium atrovenetum* and *Exophiala* sp. The last group was soils from crop rotation (CR_BS_ and CR_F_) with the dominant species *Sordariales* sp. (Figure 6).

**Figure 8:**
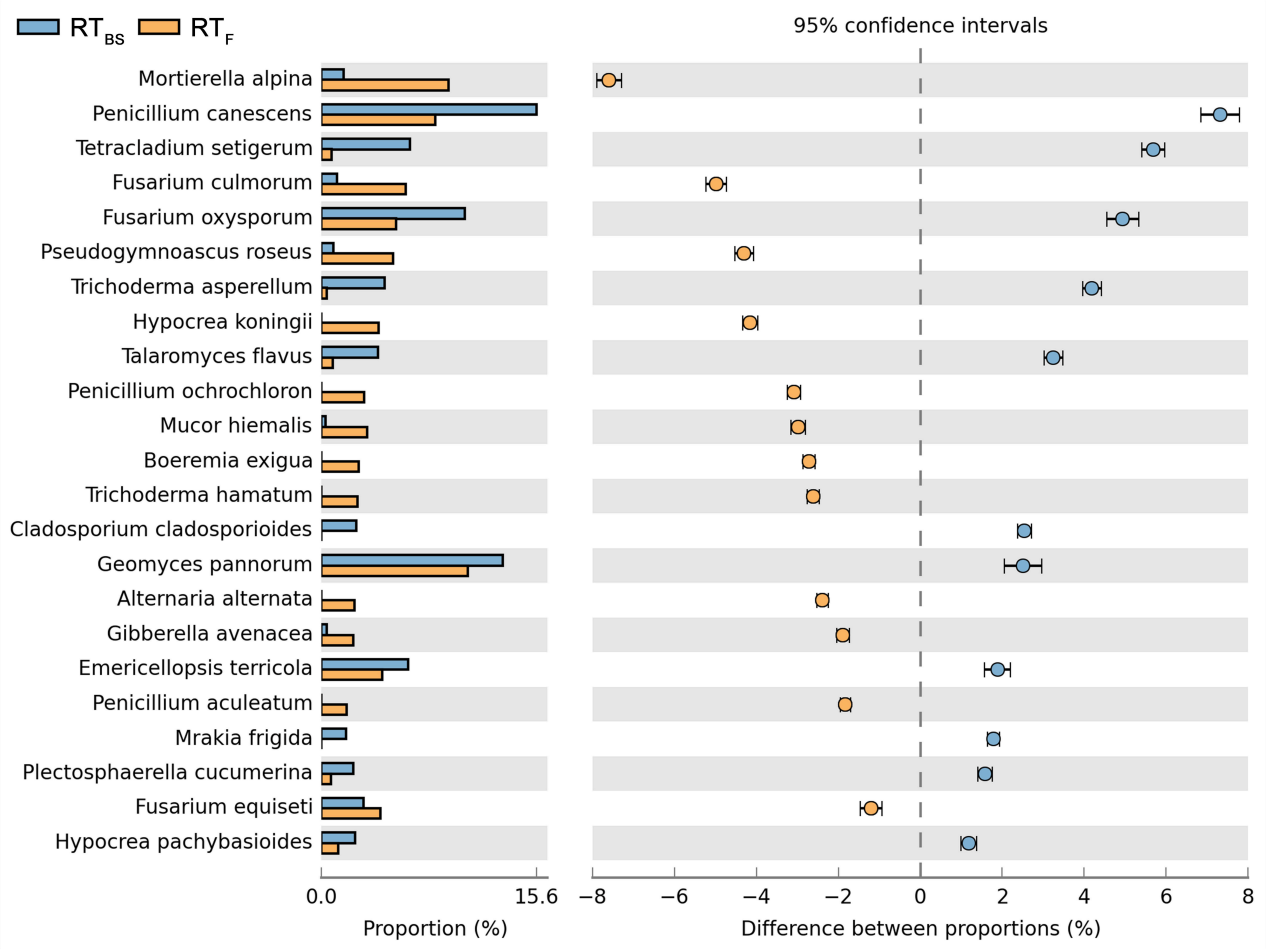
Comparison of fungal species composition depending on the season: before sowing, flowering stage and reduced tillage. The table of main species along with the full name according to **Index Fungorum** was included in Supplementary materials (Table S1).

**Figure 9:**
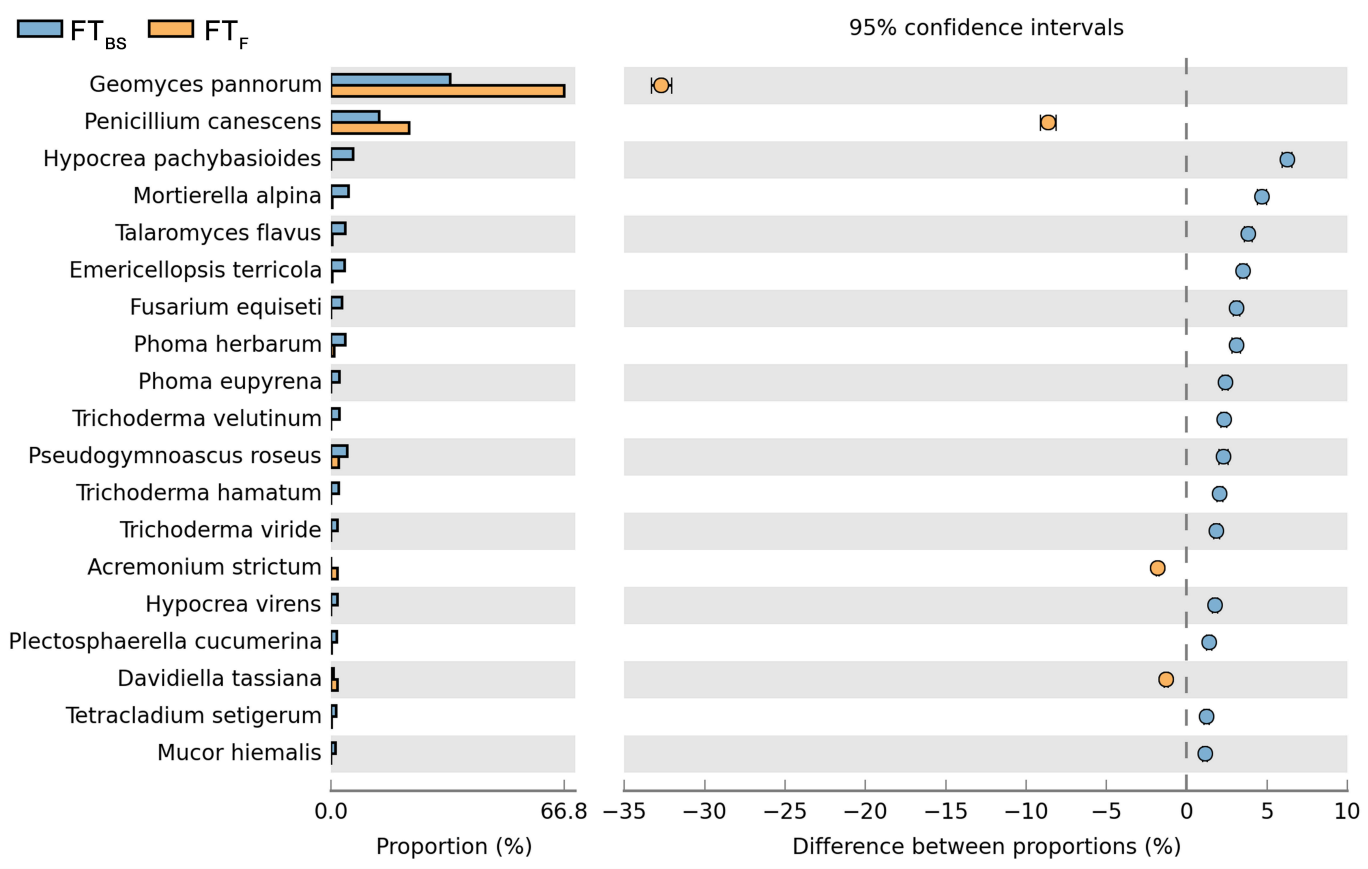
Comparison of fungal species composition depending on the season: before sowing, flowering stage and full tillage. The table of main species along with the full name according to **Index Fungorum** was included in Supplementary Materials (Table S1).

**Figure 10:**
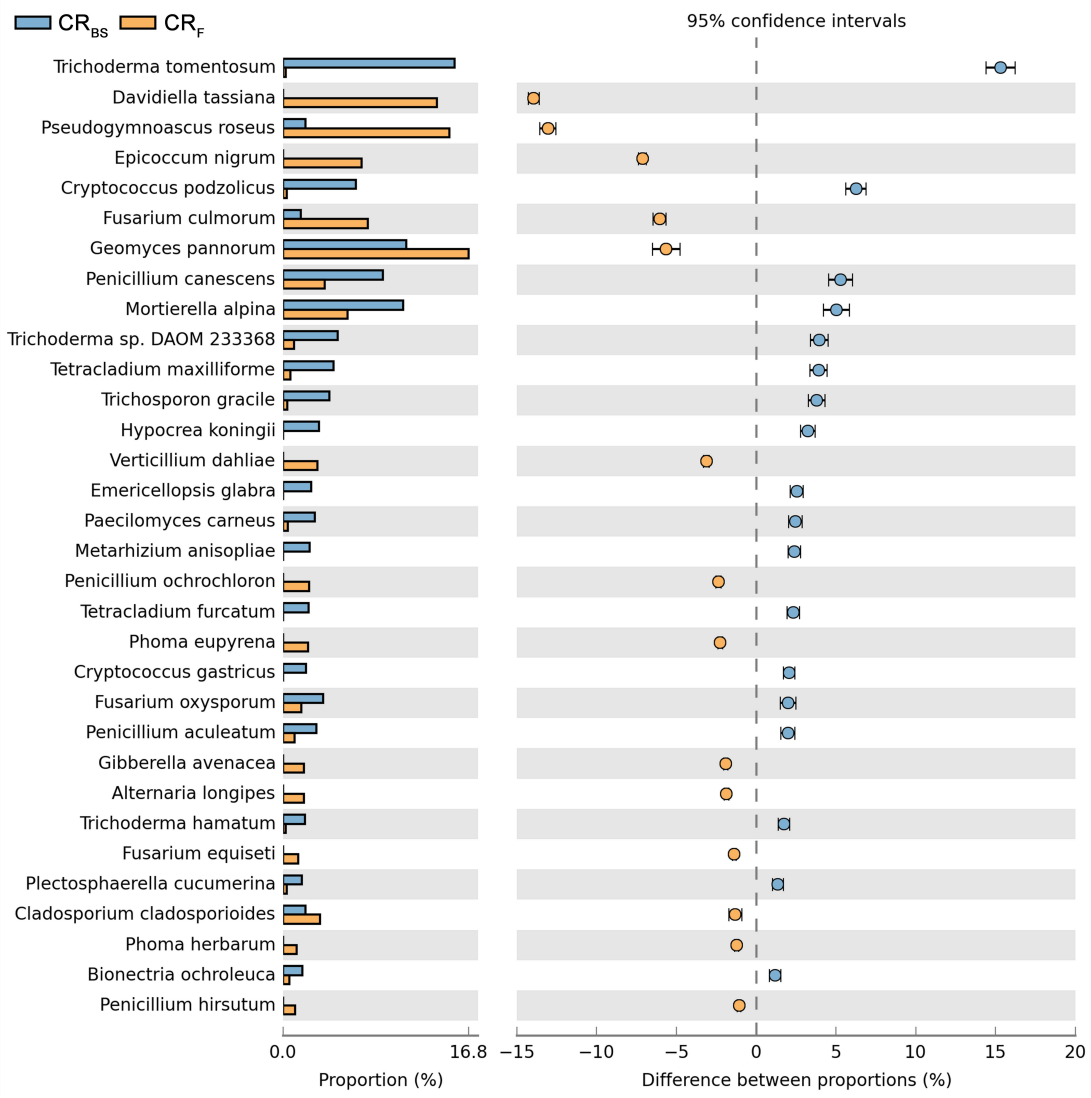
Comparison of fungal species composition depending on the season: before sowing, flowering stage and crop rotation. The table of main species along with the full name according to **Index Fungorum** was included in Supplementary Materials (Table S1).

Both cultivation techniques and the sampling time had a great influence on the fungal community in the soil. A comparison of fungal species composition, depending on the seasons (before sowing, in the flowering stage) and cultivation techniques, is presented in Figures 7–10. Some fungal species dominated in the soils before sowing, others in the flowering phase of maize. The highest relative abundances of *Penicillium canescens, Verticillium dahlia, Paecilomyces carneus*, and *Hypocrea koningii* was observed in the soils from direct sowing collected before sowing of maize (Figure 7). But the highest relative abundances of other species such as *Epicoccum nigrum, Davidiella tassiana, Geomuyces pannorum, Trichoderma hamatum*, and *Penicillum coprophilum* were observed in the soils collected from direct sowing at the flowering stage of maize growth (Figure 7). The soils collected from reduced tillage were characterized by the dominant species at the flowering stage such as: *Penicillium ochrochloron, Mucor hiemalis, Boeremiaexigua, Penicillium aculeatum, Trichoderma hamatum* (Figure 8). But in soils sampled before sowing, other species were dominant: *Penicillium canescens, Geomyces pannorum, Phoma herbarum*, and *Emericellopsis terricola* (Figure 8). Similar differences were observed in the soils collected from full tillage (Figure 9) and crop rotation (Figure 10). The soils from full tillage were characterized by the highest relative abundance contents of fungal species with main dominant species in the soil taken before sowing such as: *Mortierella alpine, Tataromyces flavus, Emericellopsis terricola, Phoma herbarum*, and *Trichoderma viride* (Figure 9).

## Discussion

In our study, the soils taken before the sowing of maize were characterized by statistically lower indexes of biological activity of substrate utilization than the soil collected at the flowering stage of maize. The highest Shannon—Weaver index was found in the soil from full tillage in both sampling times: before the sowing and flowering stages of maize growth, but in contrast, the lowest diversity was recorded for the soil collected before sowing from direct sowing. Other authors suggest that the microbial community, especially enzymatic activities is comparatively active in the flowering stage, while before sowing, in spring there is a decrease in their activity (Nannipieri et al., 2003; Brussaard et al., 2007; Bowles et al., 2014; Gajda et al., 2016). This hypothesis was probably connected with the quantitative composition of different root exudates of plants (Fisher et al., 2012; Baetz and Martinoia, 2014). The plant root exudates are very rich in various substances such as aminoacids, hydrocarbons, vitamins, organic acids and enzymes (Liang et al., 2011; Razavi et al., 2016). These substances may inhibit or stimulate the growth and development of fungi. On the other hand, changes in the availability of different nutrient compositions coming from different sources such as dead plant cells, root exudates and plants metabolites may have a significant effect on the selection of main fungal species and change their function (Spedding et al., 2004). In our study, the highest Average Well-Color Development (AWCD) index was observed in the soils obtained in the flowering stage of maize growth cultivated using different sowing techniques. The soil community level physiological profiling is not only connected with the plant species but also depends on the amount of plant remains from the root system (Zhang et al., 2012; Gałązka et al., 2017a). Biolog EcoPlate is the very sensitive method used by many authors for indicating even small changes in microbial structure in the soil under the influence of different abiotic and biotic factors (Frac et al., 2012; Wang et al., 2017).

The current paper focuses on the evaluation of the genetic diversity of fungi against the background of the general functional diversity in soil. The authors have choosen the Biolog EcoPlates for research, instead of specialty plates, dedicated to fungi identification (FF or SF). The Biolog EcoPlates indicate even slight changes in microbial structure in the soil under the influence of different abiotic and biotic factors. Many authors confirm that the results of functional diversity obtained from comparison of functional activity in soil with the use of the Biolog EcoPlates, FF-Plates and SF-Plates may be significantly different (Preston-Mafham et al., 2002; Klimek and Niklinska, 2007). In opinion of other authors the Biolog SF-N plates help to avoid the toxicity of TTC dye (present in the EcoPlates) to fungi (Deacon et al., 2006). In current paper, the intention of the authors was to assess only the genetic diversity of fungi against the background of functional diversity of microbial communities in soils under long-term monoculture of maize using different cultivation techniques. That's way community-level physiological profiling (CLPP) method with Biolog multiwell plates was chosen to evaluation functional diversity of microbial communities.

The post-harvest residues in direct sowing positively affect soil properties (Zhang et al., 2012; Wang et al., 2017). Also, a plant cultivation technique is a very important factor that can influence the biochemical activity of soil, and fungal diversity. In the opinion of many authors, plant cultivation in a long-term monoculture and intensive cultivation have a negative impact on the soil quality and can cause changes in the structure of the bacterial and fungal communities (Rice and Gowda, 2011; Wang et al., 2017). Plant cultivation in permanent monoculture is accompanied by a one-sided exhaustion of nutritive components, as well as by changes in the functional and genetic structure of soil microorganisms (Wang et al., 2009).

The results of our study indicate that the main group of fungi accounted for three phyla: *Zygomycota, Basidiomycota* and *Ascomycota*, while six dominant fungal classes identified as: *Dothideomycetes, Eurotiomycetes, Leotiomycetes, Pezizomycetes, Tremellomycetes*, and *Mortierellomycotina*. *Ascomycota* was the dominant phylum identified in all the analyzed soil samples. Our results are consistent with the results of other authors (Ma et al., 2013). The soils collected from full tillage in summer at the flowering stage of maize were characterized by the highest diversity in the genetic structure of the fungal community.

Klaubauf et al. (2010) investigated fungal diversity in four arable soils and one grassland in Lower Austria. According to the results of their research all soils were dominated by the ascomycetous orders *Sordariales, Hypocreales*, and *Helotiales*, taxa that are known from conventional cultivation approaches occurring in agricultural soils (Klaubauf et al., 2010). Our results also confirm these relationships.

On the other hand, a higher fungal genetic diversity in the soil collected from full tillage indicates that the maize is a very good plant to be grown in monoculture due to the large amount of root exudates into the soil (Baetz and Martinoia, 2014). Han et al. (2017) have proven how biotic and abiotic factors affected fungal diversity in the soil. This study raises questions about the important roles and ecological implications of fungal diversity associated with plant and environmental factors. According to these results compared with edaphic properties controlling soil fungal community patterns, the plant growth stage was the dominant factor in determining their dynamics and development.

The differences in fungal genetic communities in soils under long-term monoculture using different cultivation techniques were significant and caused characteristic changes in the structure of the fungal population (Lupwayi et al., 1998; Brussaard et al., 2007; Bowles et al., 2014; Ghimire et al., 2014). In our study, there was a negative impact observed on community level physiological profiling and fungal genetic structure in the soil under maize cultivated in long-term monoculture and full tillage. But, on the other hand, maize cultivated in direct sowing did not cause negative changes in the fungal structure making it more stable even during seasonal changes.

Direct sowing and reduced tillage are very good cultivation techniques for the development of the some group of fungi, especially mycorrhizal fungi. The large amount of crop residues left on the soil surface and slight interference with the soil structure may beneficially affect the stability of hyphae in the soil. In full tillage, the fungal hyphae are torn off, which significantly reduces their strangeness in the soil (Gianinazzi et al., 2010). In our study, the order *Glomerales* (belonging to mycorrhizal fungi) was identified in soils collected from direct sowing and reduced tillage. The order *Glomerales*, which includes the family *Glomeraceae* with genus the *Glomus*, is the very important group of arbuscular mycorrhizal fungi (AM) (Ngosong et al., 2010; Gałązka et al., 2017b). AM fungi participate in the bilateral exchange of carbon, phosphorus and other physiologically significant particles. These fungi also have the ability to produce and store in fungal filaments, the special fungal glycoproteins (glomalins) (Rillig, 2004). The carbon present in the glomalins has a large share of the organic carbon in the structure (Gianinazzi et al., 2010). In the natural environment, there is a large diversity of AM fungi (Ngosong et al., 2010). No other fungi, except for *Glomeromycota*, produce glomalins in a significant quantity.

Other genera that are very common in the soils collected from direct sowing and reduced tillage are *Fusarium* and *Penicillium*. Species of *Penicillium* are ubiquitous soil fungi preferring a moderate climate, commonly present wherever organic material is available (Duniere et al., 2017). *Fusarium* is commonly found in the soil and on underground and above - ground parts of plants, including seeds, plant residues and other organic substrates (Leslie and Summerell, 2013). *Fusarium* is common in temperate and tropical climate zones. This genus includes saprotrophic species and economically important species that are pathogenic for plants, causing significant economic losses in cereals (McMullen et al., 2012; Salgado et al., 2015). Of particular importance is the ability of *Fusarium* to produce a variety of toxins, very harmful to humans and animals, which contaminate plant foods with feed and food. *Fusarium* also produces phytotoxins that inhibit the growth of infected plants or cause their wilt, mainly seedlings (Salgado et al., 2015). They can also alter the metabolism of plants in an unfavorable way and act as virulence factors.

## Conclusions

The main research objective presented in this paper involved examination of fungal structural diversity in the soil. The results of this study have contributed to a better understanding of genetic diversity and the composition of the population of fungi in the soil environment under the influence of the changes that take place in the soil under long-term maize cultivation. The cultivation techniques can modify the fungal community in the soil under long-term monoculture of maize. Maize cultivated in direct sowing did not cause negative changes in the fungal structure, making it more stable even during seasonal changes. The biggest changes in the fungal community were observed in full tillage of maize cultivation. In this study, soil under direct sowing and reduced tillage treatment had a higher biological activity, based on community level physiological profiling (especially in the soil samples collected from the flowering stage of maize growth), than that under full tillage and crop rotation treatment. The fungal genetic structure was significantly correlated with agricultural practices and seasons. Agricultural practices and seasons were two important factors affecting the fungal community. Further research is needed to determine the function of the main genera and species dominant in soil under monoculture of maize and to identify key organisms and their dynamics under maize growth using different agricultural management practices. Also, further studies need to quantify the fungal effects on soil biochemical cycling of nutrients and maize production. It is also necessary to conduct further studies on the bacterial identification and comparison of the bacterial and fungal relationship in the soil from long-term monoculture of maize.

## Author contributions

AG: Contributions to the conception of the experiment; AG: Contributions to the design of the experiment; AG, JG: The acquisition and analysis of the data; AG, JG: The interpretation of data for the work; AG, JG: Drafting and writing the work; AG, JG: Revising the manuscript; AG, JG: Final approval of the manuscript and agreement to be accountable for all aspects of the work.

### Conflict of interest statement

The authors declare that the research was conducted in the absence of any commercial or financial relationships that could be construed as a potential conflict of interest.
