# Supplementary material for: Fungal Genetics and Functional Diversity of Microbial Communities in the Soil under Long-Term Monoculture of Maize Using Different Cultivation Techniques
Source: Front Microbiol. 2018 Jan 30;9:76. doi: 10.3389/fmicb.2018.00076 (PMC5797640; doi:10.3389/fmicb.2018.00076)
Supplement: Supplementary file 3 [file Table1.DOCX]

**TABLE 1** Classification of rate summary (at least 97% sequence similarity).

| Level | DS_BS_ | | RT_BS_ | | FT_BS_ | | CR_BS_ | |
| --- | --- | --- | --- | --- | --- | --- | --- | --- |
|  | Reads classified | % reads classified | Reads classified | % reads classified | Reads classified | % reads classified | Reads classified | % reads classified |
| Total | 152043 | 67.74% | 237924 | 72.38% | 161817 | 69.66% | 60746 | 44.01% |
| Kingdom | 151062 | 67.30% | 236579 | 71.97% | 161299 | 69.44% | 60370 | 43.74% |
| Phylum | 116773 | 52.03% | 183451 | 55.81% | 113004 | 48.65% | 44871 | 32.51% |
| Class | 92230 | 41.09% | 139694 | 42.50% | 87925 | 37.85% | 36056 | 26.12% |
| Order | 88550 | 39.45% | 132700 | 40.37% | 81183 | 34.95% | 34082 | 24.69% |
| Family | 77071 | 34.34% | 114653 | 34.88% | 66590 | 28.67% | 26539 | 19.23% |
| Genus | 68283 | 30.42% | 100732 | 30.64% | 59943 | 25.81% | 23254 | 16.85% |
| Species | 152043 | 67.74% | 237924 | 72.38% | 161817 | 69.66% | 60746 | 44.01% |
| Level | **DS_F_** | | **RT_F_** | | **FT_F_** | | **CR_F_** | |
|  | Reads classified | % reads classified | Reads classified | % reads classified | Reads classified | % reads classified | Reads classified | % reads classified |
| Total | 195435 | 70.17% | 191865 | 71.41% | 197008 | 65.94% | 162114 | 67.51% |
| Kingdom | 194772 | 69.94% | 191108 | 71.13% | 196808 | 65.87% | 160723 | 66.93% |
| Phylum | 166591 | 59.82% | 153978 | 57.31% | 147680 | 49.43% | 125955 | 52.45% |
| Class | 131072 | 47.06% | 126837 | 47.21% | 138063 | 46.21% | 102494 | 42.68% |
| Order | 127966 | 45.95% | 122951 | 45.76% | 136105 | 45.55% | 98775 | 41.13% |
| Family | 109375 | 39.27% | 106423 | 39.61% | 125876 | 42.13% | 82525 | 34.36% |
| Genus | 97754 | 35.10% | 95722 | 35.63% | 123144 | 41.22% | 71722 | 29.87% |
| Species | 195435 | 70.17% | 191865 | 71.41% | 197008 | 65.94% | 162114 | 67.51% |

Taxonomy: UNITE version 7, release date 2nd March 2015, modified

soil taken before sowing: DS_BS_ – direct sowing, RT_BS_-reduced tillage, FT_BS_-full tillage, CR_BS_-crop rotation,

soil taken in flowering stage of maize growth : DS_F_ – direct sowing, RT_F_-reduced tillage, FT_F_-full tillage, CR_F_-crop rotation,
